# Development of Deferoxamine‐Functionalized Cyclodextrin Polymer for Targeted Iron Chelation

**DOI:** 10.1002/open.202500186

**Published:** 2025-04-07

**Authors:** Roberta Panebianco, Maurizio Viale, Graziella Vecchio

**Affiliations:** ^1^ Dipartimento di Scienze Chimiche Università degli Studi di Catania Viale A. Doria 6 95125 Catania Italy; ^2^ U. O. Bioterapie IRCCS Ospedale Policlinico San Martino L.go R. Benzi, 10 16139 Genova Italy

**Keywords:** cancer, carbohydrates, chelation, desferal, nanoparticles

## Abstract

Polymeric systems are used in clinical settings to bind small molecules and ions. They show advantages in pharmacokinetics and reduced side effects compared to low‐molecular‐weight agents. In this study, a cross‐linked β‐cyclodextrin polymer is functionalized with deferoxamine to develop more efficient ways of delivering iron chelators. Deferoxamine is a well‐known iron chelator used to treat iron overload conditions. The resulting deferoxamine polymeric conjugate, with an equal complexing capacity to deferoxamine, showed significantly lower cytotoxicity compared to free deferoxamine in all cancer cell lines studied (neuroblastoma SHSY5Y, breast cancer MDA‐MB‐231, ovarian adenocarcinoma A2780, and lung cancer A549). The results demonstrate that the conjugation reduces the toxicity of deferoxamine at the same complexing capacity and reveals the potential of deferoxamine‐based cyclodextrin conjugates as a novel approach for targeting iron dyshomeostasis.

## Graphical abstract

The cyclodextrin polymer is conjugated with 35 deferoxamine units and can form iron(III) complexes. The polymer exhibits significantly lower antiproliferative activity than free deferoxamine. The cyclodextrin polymer with multiple deferoxamine units may present a promising strategy for improving the efficacy of iron chelation therapies.

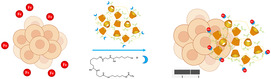

## 1 Introduction

Nanotechnologies have revolutionized the biomedical field, leading to significant advancements in developing functional nanomaterials for a wide range of clinical applications.^[^ ^1^ ^]^ Conventional small‐molecule drugs are hindered by several limitations, including premature degradation, rapid clearance from the body, limited ability to traverse biological barriers, nonspecific distribution, and undesirable side effects. Nanoscale systems have been developed to mitigate these issues, leveraging their small size, high surface‐to‐volume ratio, and favorable characteristics for drug release, which can enhance the pharmacokinetic and pharmacodynamic properties of therapeutic compounds.^[^ ^2^ ^]^

Generally, various nanosystems, including polymers, hybrid nanoparticles, micelles, liposomes, dendrimers, and carbon nanotubes, have been studied.^[^ ^3^, ^4^, ^5^, ^6^, ^7^ ^]^ The suitability of these nanomaterials for applications within the human body hinges on their bioactivity, mechanical properties, degradation rates, and biocompatibility.^[^ ^8^ ^]^

Polymeric nanoparticles (PNPs) are among the most effective materials for biological applications since they can be tailored to carry drugs, targeting ligands, and imaging agents. They can confer several properties to the linked molecules, such as excellent solubility, specific targeting capacity, high accumulation at tumor sites, and reduced side effects.

In general, polymeric systems are widely used in clinical applications to bind small molecules and ions, such as phosphate and potassium.^[^ ^9^ ^]^ These polymeric sequestrants enhance the pharmacokinetics and mitigate side effects compared to small‐molecule sequestrants. Sodium polystyrene sulfonate (Kayexalate) was the first synthetic polymeric sequestrant used to treat hyperkalemia.^[^ ^10^ ^]^ Polyamine polymers, such as Sevelamer (trade name Renagel), have also found therapeutic applications through oral administration as phosphate binders in the treatment of hyperphosphatemia associated with kidney diseases.^[^ ^11^ ^]^ Colesevelam hydrochloride (WelChol) has been used as a bile acid binder to reduce cholesterol levels.^[^ ^12^ ^]^

Polysaccharide polymers provide a range of advantages that make them attractive materials for developing polymer–drug conjugates, enhancing the efficacy and safety of drug delivery systems.^[^ ^13^, ^14^ ^]^ Additionally, polysaccharides are biodegradable. This property allows for rapid clearance after drug delivery and diminished toxicity.^[^ ^15^ ^]^

The hydrophilic nature of polysaccharides enhances the solubility of the polymer–drug conjugate in biological fluids, thereby improving bioavailability and facilitating the drug more efficient absorption by the body. Furthermore, polysaccharides generally exhibit low immunogenicity, even after repeated administrations. Another advantage of polysaccharides is their functionalizable structure. Their reactive groups can be modified, allowing smart drug delivery to specific tissues or organs.

As a result, in recent years, efforts have been focused on utilizing these advantages across the pharmaceutical field.^[^ ^16^, ^17^, ^18^ ^]^

Among carbohydrates, cyclodextrins (CDs) are particularly appealing carriers in drug delivery systems, owing to their biocompatibility and distinctive chemical structure.^[^ ^19^ ^]^ They have appealing applications in numerous fields, including supramolecular chemistry, bioinorganic chemistry, organic chemistry, cosmetics, pharmaceuticals, and materials science.^[^ ^20^, ^21^, ^22^, ^23^, ^24^ ^]^ CDs have also been investigated for colon‐targeted delivery because they are resistant to gastric acid and enzymatic degradation, and can reach the colon intact.^[^ ^25^, ^26^ ^]^ Cross‐linked, linear, or branched CD polymers (pCDs) have recently been synthesized.^[^ ^27^, ^28^, ^29^, ^30^ ^]^ CD polymers present considerable potential for utilizing cyclodextrin features in various applications.^[^ ^31^, ^32^, ^33^, ^34^ ^]^ They have been used as nanotherapeutics and excipients in drug delivery. Cross‐linked CD polymers exhibited a three‐dimensional network, and their properties depended strongly on the density of the cross‐linker. pCDs can be chemically modified with targeting units^[^ ^35^, ^36^ ^]^ drugs^[^ ^37^, ^38^, ^39^ ^]^ fluorescent moieties,^[^ ^30^ ^]^ and cationic functional groups^[^ ^29^, ^40^, ^41^, ^42^ ^]^ to introduce new properties useful for their application in biological systems, Some nano‐prodrugs based on CDs are currently in phase I and II clinical trials as drug delivery systems.^[^ ^43^ ^]^

Many families of CD polymers have been investigated for their applications in food and pharmaceutical science.^[^ ^33^, ^44^, ^45^, ^46^ ^]^ CD‐based polymers have recently been functionalized with chelators such as carcinine or histidine to obtain s copper(II) nanochelators.^[^ ^47^ ^]^ CD conjugation has been proven as a promising approach for colon targeting due to the CD ability to remain intact in gastric and intestinal regions.^[^ ^48^ ^]^

Based on the potential of polymeric formulations, the advantages of deferoxamine B (DFO) conjugation with polysaccharides have also been reported.^[^ ^49^, ^50^, ^51^ ^]^

DFO is a naturally occurring siderophore synthesized by some bacteria, often alongside other siderophores, to capture Fe^3+^ from the environment. Then, the complex siderophore‐Fe^3+^ is transported into bacteria cells through specific recognition pathways involving iron uptake proteins.^[^ ^52^ ^]^ The high affinity of DFO for Fe^3+^ derives from its structure, which contains three bidentate hydroxamic functional groups that form Fe^3+^ octahedral complexes with a high stability constants.^[^ ^52^ ^]^

The ability of DFO to bind Fe^3+^ and various metal cations,^[^ ^53^ ^]^ along with the abundance of data on its biological activity, toxicity, long‐term effects on the human body, acceptable water solubility, low molecular weight, and relatively low cost, has determined its application in numerous fields. DFO is used in iron chelation therapy (Desferal) to treat diseases and conditions related to iron ion imbalances.^[^ ^54^ ^]^ Iron chelators have been shown to slow down cancer cell growth by inducing cell cycle arrest and apoptosis, or by targeting mitochondrial functions.^[^ ^55^, ^56^, ^57^, ^58^, ^59^ ^]^

The development of DFO conjugates is facilitated by the terminal amino group, which is not involved in metal coordination and can be easily linked to small molecules, antibiotics, antibodies, or other biologically significant compounds, enabling the design of derivatives with new properties.^[^ ^51^ ^]^ A promising approach to enhance its pharmacokinetics involves covalently conjugated DFO to biocompatible polymers via its terminal amino group.^[^ ^50^, ^51^ ^]^

We hypothesized that conjugating DFO to a cyclodextrin polymer would preserve its iron‐chelating capacity while altering its cytotoxicity due to the properties of the polymer. In particular, CD ability to retain in the GI tract can be exploited to target the iron chelation in the duodenum and improve the efficiency of the chelation to reduce dietary iron absorption. A key advantage of the polymeric formulation of chelators is the potential for oral administration compared to the intramuscular delivery required for DFO.

**Figure 1:**
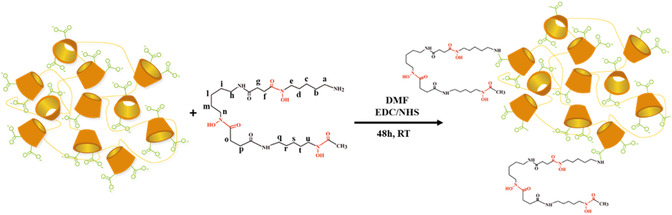
Synthetic scheme of the cross‐linked β cyclodextrin polymer–deferoxamine conjugate (pβCD‐DFO). Only two of the 35 DFO units have been sketched for simplicity.

In this study, we synthesized a new cross‐linked β‐cyclodextrin polymer conjugated with DFO (pβCD‐DFO, **Figure** **1**). DFO was linked by the amino group, which is not involved in iron(III) coordination^[^ ^52^ ^]^ to preserve the coordination properties of the ligand units in the CD polymer. The polymer conjugated with DFO was characterized by NMR spectroscopy and dynamic light scattering (DLS). We also examined its iron‐chelating capability with UV‐Vis spectroscopy. We evaluated the toxicity of pβCD‐DFO alone and in the presence of iron(III) in A2780 (ovary, adenocarcinoma), A549 (lung, adenocarcinoma), SHSY5Y (neuroblastoma) and MDA‐MB‐231 (breast, adenocarcinoma) cancer cells in comparison to DFO.

The conjugate pβCD‐DFO containing 35 DFO units can form iron(III) complex. Our results demonstrated that the conjugation reduced the toxicity of DFO in cancer cells referred to as DFO units and revealed the potential of DFO‐based CD conjugates as a novel approach for targeting iron dyshomeostasis.

## 2 Results and Discussion

### 2.1 Synthesis and Characterization

DFO was conjugated to a cross‐linked β‐cyclodextrin polymer of 84 kDa containing carboxylic acid groups (pβCDCOOH) through a condensation reaction between the primary amino group of DFO and the carboxylic groups of the polymer using the EDC/NHS method, as illustrated in Figure 1. The pβCD‐DFO conjugate was isolated by dialysis against MilliQ water.

**Figure 2:**
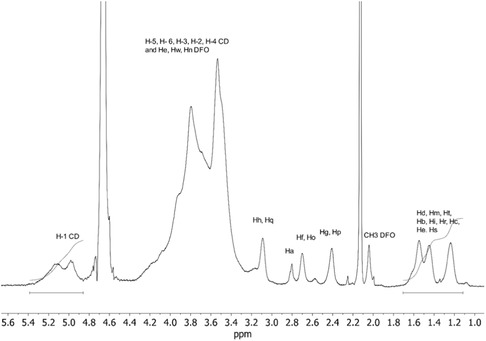
^1^H NMR spectrum of pβCD‐DFO (500 MHz, D_2_O) (signal of acetone is in the spectrum at 2.1 ppm).

The functionalization of pβCDCOH with DFO was confirmed by NMR spectroscopy (**Figure** **2** and S1–S3, Supporting Information). In the ^1^H NMR spectrum of pβCD‐DFO (Figure 2), the signals are broad, as typically observed for other pβCD conjugates. Hs‐1 of CDs resonate in the range between 5.3 and 4.8 ppm, whereas the other signals of the CDs are observed at a higher field (*δ* = 4.08–3.35 ppm). Diagnostic peaks of DFO moieties are present between 3.2 and 1.2 ppm, with the CH_3_ group at 2.04 ppm, and other CH_2_ groups overlapping with H‐2 and H‐4 of CDs. In the spectra, the small signal at 2.57 ppm is due to the Z isomer of hydroxamic group equilibrium, as reported for free DFO.^[^ ^60^ ^]^ By integrating the Hs‐1 signal of CDs and the characteristic proton signals of DFO units at *δ* = 1.6–1.2 ppm, the degree of functionalization was calculated to be 65% of CD units. This corresponds to an average of 35 DFO molecules conjugated to pβCDCOOH polymer.

These results confirmed the high degree of functionalization of the native CD polymer. The molecular weight of pβCD‐DFO is about 103 kDa.

### 2.2 Iron Complex Characterization: UV‐Vis Spectroscopy

DFO has three hydroxamic groups with high affinity for the Fe^3+^ ion. Numerous studies on the solution equilibria of the DFO/Fe^3+^ system have been reported in the literature.^[^ ^52^ ^]^ These studies confirm the formation of a 1:1 complex under equimolar metal/ligand and also in highly acidic conditions. The predominant species in the pH range of 1–10 is [FeH(DFO)]^+^, where the amino group is protonated, with a logβ of ≈41.5. At pH values outside this range, the main species are [Fe(DFO)] with a logβ of around 31 at higher pH and [FeH_2_(DFO)]^2+^ species with a logβ of about 42.5 at lower pH.^[^ ^52^ ^]^ The complex of DFO and Fe^3+^ exhibits a characteristic absorption peak of around 430 nm due to a ligand‐to‐metal charge transfer (LMCT) transition. The formation of the Fe^3+^ complex imparts a yellow‐brown color to the solution.^[^ ^61^ ^]^

**Figure 3:**
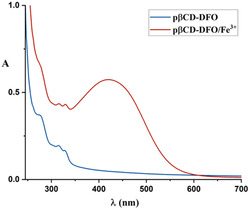
UV‐Vis spectra of pβCD‐DFO (blue line) and pβCD‐DFO/Fe^3+^ (red line).

**Figure 4:**
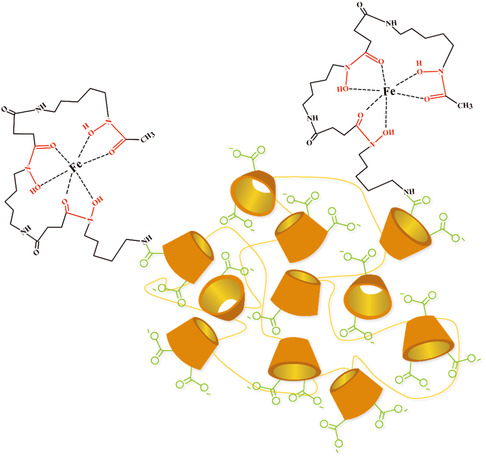
Schematic structure of pβCD‐DFO/Fe^3+^ complex. Only two of the 35 DFO units have been sketched for simplicity.

To elucidate the iron‐chelating ability of pβCD‐DFO, the UV‐Vis absorption spectra were acquired in the absence and presence of Fe^3+^ in a 35:1 Fe^3+^/pβCD‐DFO molar ratio. The DFO amino group functionalized in the polymers is not involved in the iron coordination. Similarly to the DFO, the conjugate pβCD‐DFO did not exhibit transitions in the visible region, while the complex pβCD‐DFO/Fe^3+^ showed a peak around 430 nm, as reported in **Figure** **3**, suggesting that pβCD‐DFO possessed an iron‐chelating ability comparable to that of free DFO. The schematic structure of pβCD‐DFO/Fe^3+^ is reported in **Figure** **4**.

### 2.3 DLS Studies

*Z*‐average diameter and Zeta potential of the pβCD‐DFO and pβCD‐DFO/Fe^3^^+^ systems were investigated by DLS (**Table** **1** and Figure S4, Supporting Information).

**Table 1:** Hydrodynamic diameter values (*Z*‐average), polydispersity index (PDI) and zeta potential of pβCD‐DFO and pβCD‐DFO/Fe^3+^ at pH = 7.4.

System	*Z*‐average (*d*, [nm])	PDI	Zeta potential [mV]
pβCD‐DFO	100 ± 2	0.448	−28 ± 1
pβCD‐DFO/Fe^3+^	87 ± 3	0.336	−20 ± 2

The pβCD‐DFO average particle size is ≈100 nm, whereas the *Z*‐average size decreases to around 87 nm upon complexation with Fe^3^^+^. This slight size reduction may indicate tighter conformation of the conjugate upon metal ion binding due to the rearrangement of pβCD‐DFO induced by the coordination of Fe^3^^+^ ions. Also, the DLS curves in Figure S4, Supporting Information, displayed a single peak, indicating that the resulting nanoparticles are relatively monodisperse. The Zeta potential of pβCD‐DFO at pH 7.4 was −28 ± 1 mV, indicating that the NPs possess a moderate negative surface charge, primarily due to the ionized carboxyl groups under these pH conditions. After adding Fe^3+^, the Zeta potential is only slightly different from that of the ligand, in keeping with the formation of the neutral Fe^3+^ complex with the hydroxamate groups of the DFO units  (Figure 4).

**Figure 5:**
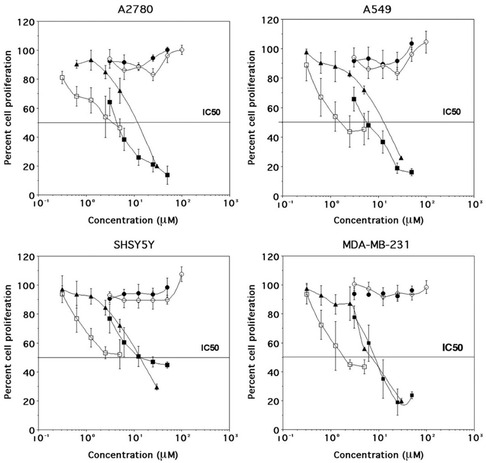
Dose–response curves for Fe^3+^ (○), DFO (▪), pβCDDFO (□), DFO with 100 μm Fe^3+^ (•), pβCDDFO with 100 μm Fe^3+^ (▴) in A2780, A549, SHSY5Y, and MDA‐MB‐231 cell lines.

The antiproliferative activity of pβCD‐DFO was evaluated in the presence of Fe^3+^ in A2780 (ovary, adenocarcinoma), A549 (lung, adenocarcinoma), SHSY5Y (neuroblastoma), and MDA‐MB‐231 (breast, adenocarcinoma). DFO and cisplatin were studied for comparison (**Figure** **5**). IC_50_ values are reported in **Table** **2**.

**Table 2:** IC_50_ values (μm) of DFO and pβCD‐DFO alone and in the presence of Fe^3+^ (100 μm).

System	A2780	A549	SHSY5Y	MDA‐MB‐231
FeCl_3_	≫30	≫30	≫30	≫30
DFO	4.9 ± 1.0	5.3 ± 1.2	12.1 ± 4.4	7.48 ± 2.2
DFO/Fe^3+^	>30	>30	>30	>30
pβCD‐DFO	3.9 ± 1.5	2.0 ± 0.9	3.1 ± 0.79	2.5 ± 1.2
pβCD‐DFO/Fe^3+^	16.1 ± 1.1	16.2 ± 4.1	19.5 ± 2.7	7.2 ± 0.9
Cisplatin	1.6 ± 0.3	6.9 ± 1.5	0.83 ± 0.12	18.2 ± 4.8

Free DFO demonstrated antiproliferative activity in vitro that was comparable to cisplatin, especially in MDA‐MB‐231 and A549 cells. These results align with numerous prior studies suggesting that iron deprivation represents a promising therapeutic strategy in cancer treatment under in vitro conditions and can induce cell cycle arrest.^[^ ^62^, ^63^, ^64^ ^]^ However, pβCD‐DFO exhibited a slightly enhanced potency compared to DFO in the A2780 and A549 cell lines, with a more pronounced increase in activity observed in SHSY5Y and MDA‐MB‐231 cells. The gain in terms of inhibition of cell proliferation is, on average, 2.7 times greater than that of DFO. If the IC_50_ of pβCD‐DFO was referred to the DFO unit concentration (35 times the polymer concentration), the toxicity of the polymer was significantly lower than that of DFO. In all cell lines, the IC_50_ was >50 μm. Upon adding Fe^3+^, a marked reduction in cytotoxicity was observed, suggesting that the antiproliferative activity of these compounds was linked to their iron‐chelating capacity and the consequent induction of iron deprivation.

However, in the presence of 100 μm Fe^3+^, DFO was pharmacologically inactive across all tested cell lines, whereas pβCD‐DFO demonstrated only a lower antiproliferative effect compared to the ligand alone. In the case of pβCD‐DFO, the concentration of DFO units is higher than Fe^3+^, which can explain the residual observed cytotoxicity. Overall, the IC_50_ values increased in the presence of Fe^3+^.

## 3 Conclusion

A cross‐linked water‐soluble β‐cyclodextrin polymer was modified with a high functionalization degree with deferoxamine B. The 35 deferoxamine moieties within the cyclodextrin polymer can effectively bind Fe^3+^. The new polymer and its iron complex form nanoparticles with a diameter of about 100 nm.

The behavior of the new β‐cyclodextrin polymer was compared with deferoxamine in cancer cells. Deferoxamine exhibited antiproliferative activity similar to that of cisplatin, and the toxicity of the polymer–deferoxamine conjugate showed an average antiproliferative activity 2.7‐fold greater than that of deferoxamine. On the contrary, when referring the IC_50_ to the deferoxamine units in the deferoxamine–polymer conjugate, the polymer with an equal complexing capacity exhibited significantly lower antiproliferative activity. This behavior suggests that the binding of deferoxamine to the polymers significantly reduced its toxicity.

The iron‐binding activity of deferoxamine plays a crucial role in the mechanism of action. Indeed, the reduction in antiproliferative effects upon adding Fe^3^^+^ supports the hypothesis that iron depletion in cells occurred and it is a crucial factor in the observed cytotoxicity of chelators. In the case of the polymer, the chelation of iron was efficient in vitro, and the observed antiproliferative activity was reduced in the presence of iron(III).

The functionalization of cyclodextrin‐based polymers with multiple deferoxamine units may present a promising strategy for improving the efficacy of iron chelation therapies.

## 4 Experimental Section

4.1

4.1.1

##### Chemicals

N‐hydroxysucinimide (NHS), 1‐Ethyl‐3‐(3‐dimethylaminopropyl) carbodiimide (EDC), and DFO mesylate were purchased from Merck. The water‐soluble polymer pβCDCOOH (84 kDa, 54 cavities) was purchased from Cyclolab. Thin layer chromatography (TLC) was performed on silica gel plates (Merck 60‐F254). Carbohydrate derivatives were detected on TLC with the anisaldehyde test.

##### NMR Spectroscopy

^1^H and ^13^C NMR spectra were recorded at 25 °C with a Varian UNITY PLUS‐500 using standard pulse programs from the Varian library. 2D experiments (COSY, HSQC, and HMBC) were acquired using 1 K data points, 256 increments.

##### Dynamic Light Scattering

DLS measurements were performed at 25 °C with a Zetasizer Nano ZS (Malvern Instruments, Oxford, UK) operating at 633 nm (He–Ne laser). The mean hydrodynamic diameter (*d*) of the NPs was determined from intensity measurement after averaging five measurements. The sample solutions were diluted in HEPES buffer (10 mm, pH = 7.4) for a final concentration of about 1.5 mm. All the solutions were prepared in ultrapure water filtered (0.2 μm).

##### UV‐Vis Spectroscopy

The iron‐binding ability of pβCD‐DFO conjugate was investigated by UV‐visible spectrophotometry. Spectra were recorded with Agilent Carry 8500 spectrophotometer. The complex of pβCD‐DFO was prepared from freshly prepared stock solutions of FeCl_3_ in HCl 2 mm. The ligand (L) pβCD‐DFO and the metal (M) were mixed at a molar ratio M/L of 35:1.

##### Synthesis of Cross‐Linked β‐CD Polymer–Deferoxamine Conjugate (pβCD‐DFO)

EDC (23 mg, 0.15 mmol) and NHS (17 mg, 0.15 mmol) were added to pβCD‐COOH (200 mg, 2.40 μmol) in 4 mL of anhydrous DMF. After reacting for ≈10 min, DFO mesylate (95 mg, 0.15 mmol) was added, and the mixture was left under stirring for 48h. The product was then isolated by dialysis against MilliQ water using a membrane with a molecular weight cut‐off (MWCO) of 20 kDa. The product was washed with acetone and dried.

^1^H NMR (D_2_O, 500 MHz) *δ* (ppm): 5.3–4.8 (m, H‐1 CD), 4.3–3.2 (m, H‐2, −3, −4, −5, −6 CD, He, Hw, Hn DFO), 3.07 (m, Hh, Hq DFO), 2.97 (m, Ha DFO), 2.70 (m, Hf, Ho DFO), 2.57 (m, Hf, Ho Z form), 2.41 (m, 4H, Hg, Hp DFO), 2.01 (s, CH_3_ DFO), 1.63–1.4 (m, Hd, Hm, Ht Hb DFO), 1.42 (m, Hi, Hr DFO), 1.29–1.15 (m, Hc, He, Hs DFO).

##### Antiproliferative Activity Assay

Human cell lines A2780 (ovary, adenocarcinoma), A549 (lung, carcinoma), SHSY5Y (neuroblastoma), and MDA‐MB‐231 (breast, carcinoma), were plated into flat‐bottomed 96‐well microtiter plates at the opportune numbers in 180 μL per well of complete media (RPMI 1640 for A2780, A549; RPMI 1640 plus 2% glutamine for SHSY5Y; DMEM for MDA‐MB‐231) added with 10% fetal bovine serum (FBS) and 1% penicillin‐streptomycin (all media and additives were obtained from EuroClone spa, Pero, MI, Italy) and also added with Fe^3+^ 100 μm when required. Plates were then centrifugated and after 6–7 h, 20 μL of normal saline containing five opportune concentrations of the compounds were administered to cells. DFO and pβCD‐DFO alone and in the presence of Fe^3+^ were studied. Plates were then processed as described elsewhere.^[^ ^65^ ^]^ The concentrations inhibiting 50% cell growth (IC_50_) were calculated based on the analysis of the concentration–response curves. Each experiment was repeated 4–6 times. IC_50_ values higher than 30 μm were considered pharmacologically irrelevant.

## Conflict of Interest

The authors declare no conflict of interest.

## Supporting information

Supplementary Material
