# Supplementary material for: Development of Deferoxamine‐Functionalized Cyclodextrin Polymer for Targeted Iron Chelation
Source: ChemistryOpen. 2025 Apr 7;14(10):e202500186. doi: 10.1002/open.202500186 (PMC12518034; doi:10.1002/open.202500186)
Supplement: Supplementary file 1 — Supplementary Material [file OPEN-14-e202500186-s001.pdf]

Development of Deferoxamine-Functionalized Cyclodextrin Polymer for Targeted Iron Chelation

Roberta Panebianco [1], Maurizio Viale [2] and Graziella Vecchio [1,*]

1. Dipartimento di Scienze Chimiche, Università degli Studi di Catania, Viale A. Doria 6, 95125 Catania, Italy; gr.vecchio@unict.it

2. IRCCS Ospedale Policlinico San Martino, U.O. Bioterapie, L.go R. Benzi, 10, 16139 Genova, Italy.

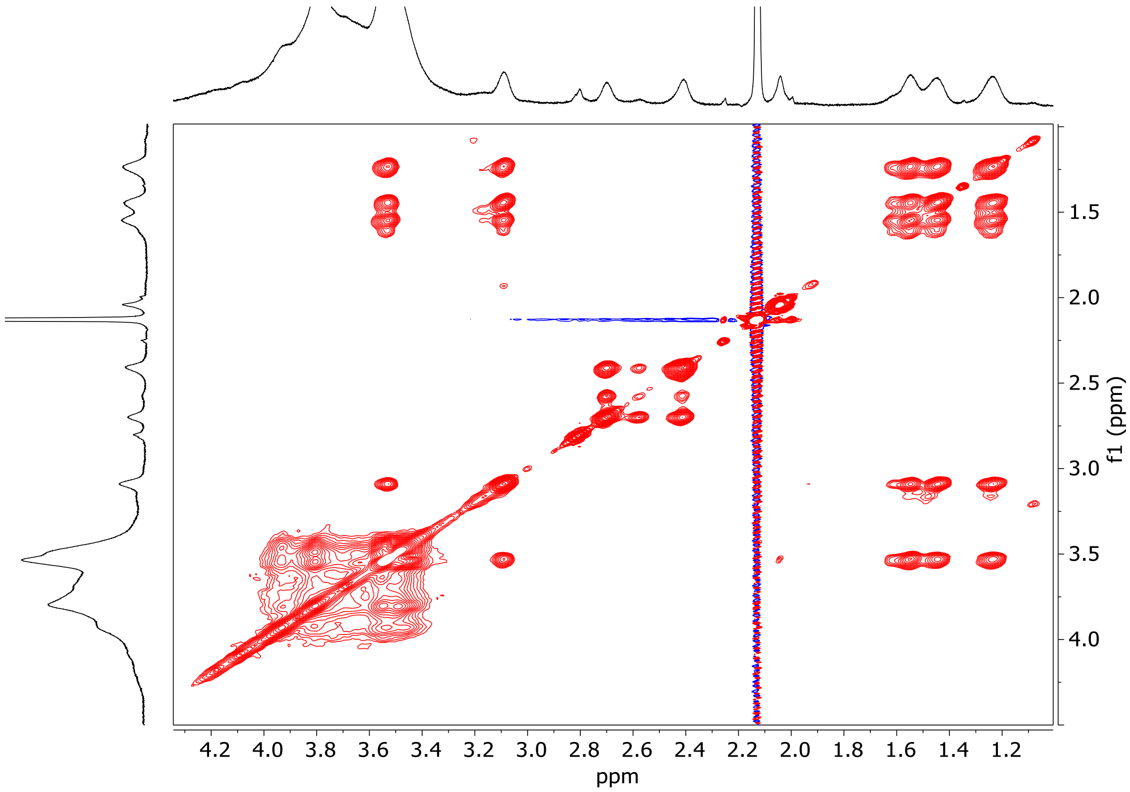

Figure 1S. COSY spectrum of pβCD-DFO (500 MHz, D_2O) (signal of acetone is in the spectrum at 2.1 ppm)

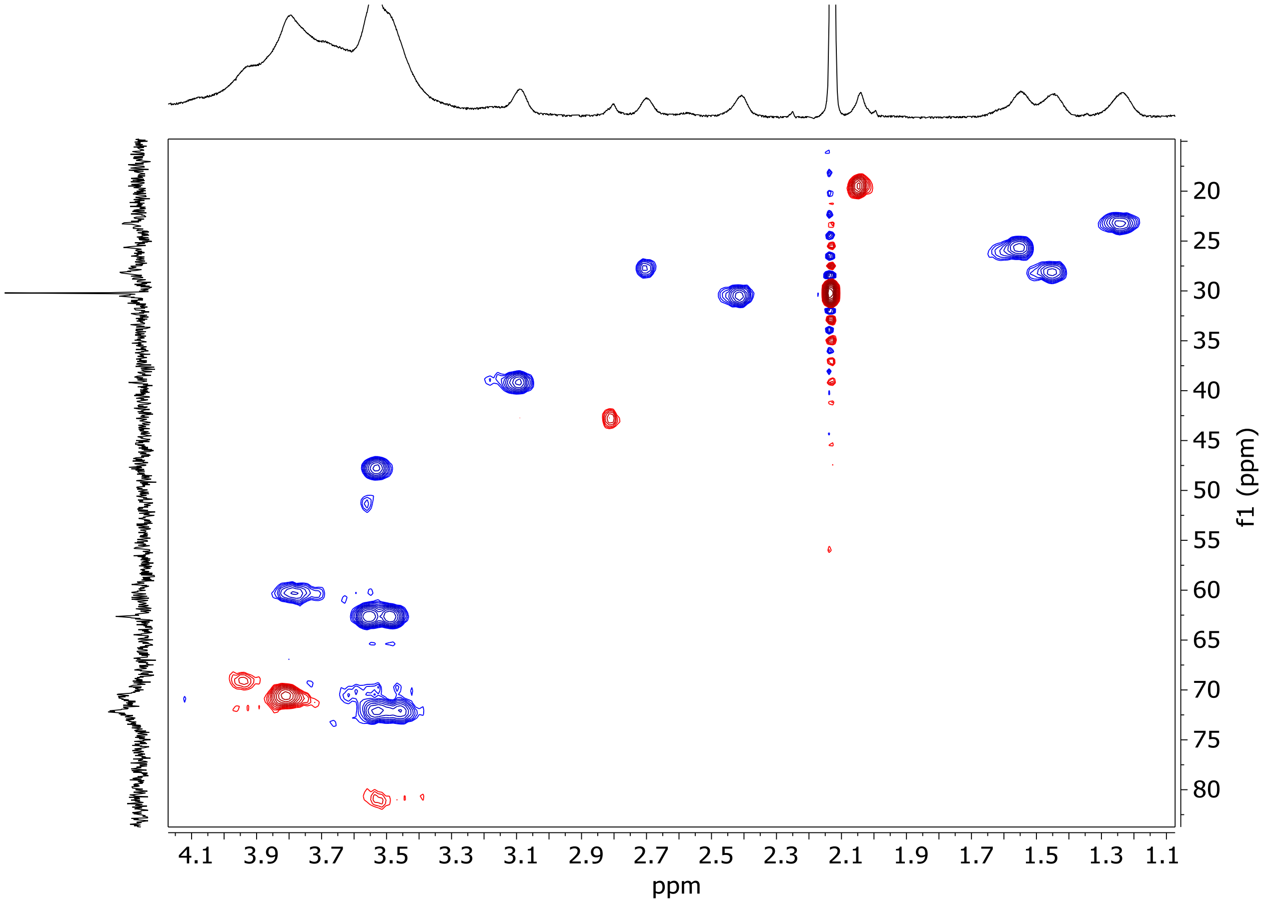

Figure 2S. HSQC spectrum of pβCD-DFO (500 MHz, D_2O) (signal of acetone is in the spectrum at 2.1 ppm)

Type your text

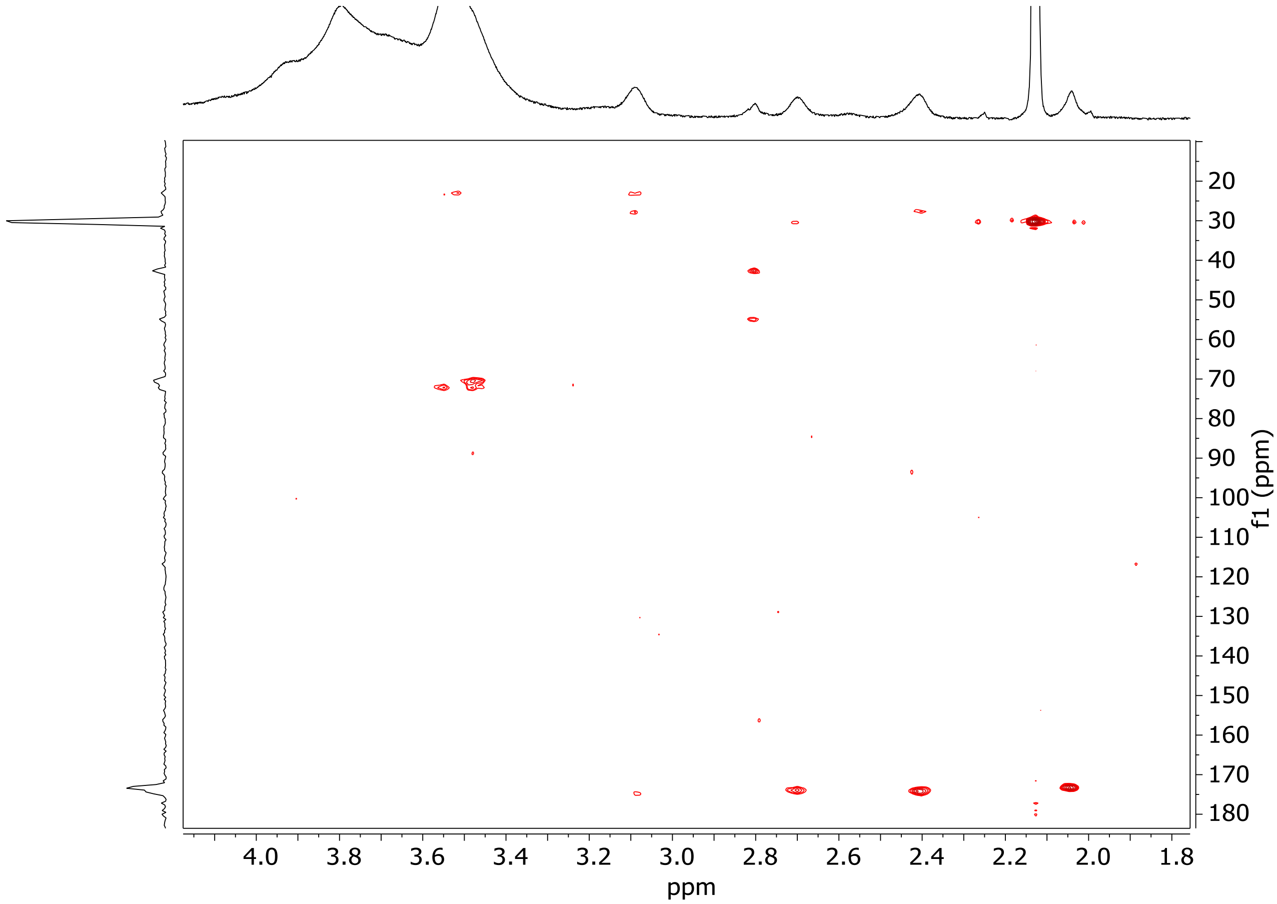

Figure 3S. HMBC spectrum (zoom) of pβCD-DFO (500 MHz, D_2O) (signal of acetone is in the spectrum at 2.1 ppm)

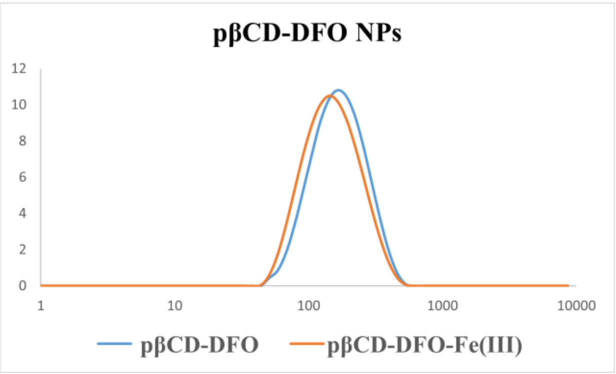

Figure 4S. DLS size distribution of pβCD-DFO and pβCD-DFO-Fe^{3+} in HEPES buffer at pH 7.4
